# "He Was Bought a Bun"

**Published:** 1917-10-20

**Authors:** 


### "He was Bought a Bun."

*To the Editor of* The Hospital.

Sir,—The question of the strict grammatical legality of the above sentence might very well be left at the point to which "B. O." carries it; for experience shows that all heterodox but convenient constructions get to be considered formally correct surprisingly soon nowadays. At school they would object to the verb *walk* being called intransitive, confronting one with the sentence "The groom walked the horse up and down the road": while they gave one to remember a long word denoting Shakespeare's use of the word *faint* in "upon faint primrose beds" (=beds of primroses upon which exhausted travellers reclined). I remember thinking the first an undignified roping-in of a colloquialism, and the second a highly factitious piece of classification. But later one sees that grammarians have to be accommodating. The construction "B. O." is good enough to notice was mainly dictated by the necessity for giving to the word conveying the point of the little joke that effective position occupied by a verb in a Latin sentence, and by the name of the leading lady in a playbill. Those of us who cannot command the liquid prose of a poet, but whose meaning has to come out in a diagrammatic form, also cannot avoid salients and twistings.—Yours faithfully,

